# *N*-(coumarin-3-yl)cinnamamide Promotes Immunomodulatory, Neuroprotective, and Lung Function-Preserving Effects during Severe Malaria

**DOI:** 10.3390/ph17010046

**Published:** 2023-12-27

**Authors:** Paulo Gaio, Allysson Cramer, Natália Fernanda de Melo Oliveira, Samuel Porto, Lucas Kramer, Rayane Aparecida Nonato Rabelo, Rafaela das Dores Pereira, Laura Lis de Oliveira Santos, César Luís Nascimento Barbosa, Fabrício Marcus Silva Oliveira, Mauro Martins Teixeira, Remo Castro Russo, Maria João Matos, Fabiana Simão Machado

**Affiliations:** 1 Department of Biochemistry and Immunology, Institute of Biological Science, Universidade Federal de Minas Gerais, Belo Horizonte 31270-901, MG, Brazil; paulogaioleite@gmail.com (P.G.); cramer.allysson@gmail.com (A.C.); melonataliaf@gmail.com (N.F.d.M.O.); samuel.porto192@gmail.com (S.P.); lucasimunologia@gmail.com (L.K.); rayane.rabelo10@gmail.com (R.A.N.R.); rafaelapereiranutri@gmail.com (R.d.D.P.); lauralis.oli@gmail.com (L.L.d.O.S.); mmtex.ufmg@gmail.com (M.M.T.); 2 Program in Health Sciences, Infectious Diseases and Tropical Medicine/Interdisciplinary Laboratory of Medical Investigation, Faculty of Medicine, Universidade Federal de Minas Gerais, Belo Horizonte 30130-100, MG, Brazil; cesarbarbosa_91@yahoo.com.br; 3 Cellular and Molecular Immunology Group, René Rachou Institute, Oswald o Cruz Foundation—FIOCRUZ, Belo Horizonte 30190-002, MG, Brazil; oliveirafms13@gmail.com; 4 Laboratory of Pulmonary Immunology and Mechanics, Department of Physiology and Biophysics, Institute of Biological Sciences, Federal University of Minas Gerais, Belo Horizonte 31270-901, MG, Brazil; russorc@gmail.com; 5 Departamento de Química Orgánica, Facultad de Farmacia, Universidade de Santiago de Compostela, 15782 Santiago de Compostela, Spain

**Keywords:** *Plasmodium berghei* ANKA, *N*-(coumarin-3-yl)cinnamamide, immunoregulation, cerebral malaria, acute respiratory distress syndrome

## Abstract

*Plasmodium berghei* ANKA (PbA) infection in mice resembles several aspects of severe malaria in humans, such as cerebral malaria and acute respiratory distress syndrome. Herein, the effects of *N*-(coumarin-3-yl)cinnamamide (M220) against severe experimental malaria have been investigated. Treatment with M220 proved to protect cognitive abilities and lung function in PbA-infected mice, observed by an object recognition test and spirometry, respectively. In addition, treated mice demonstrated decreased levels of brain and lung inflammation. The production and accumulation of microglia, and immune cells that produce the inflammatory cytokines TNF and IFN-γ, decreased, while the production of the anti-inflammatory cytokine IL-10 by innate and adaptive immune cells was enhanced. Treatment with M220 promotes immunomodulatory, neuroprotective, and lung function-preserving effects during experimental severe malaria. Therefore, it may be an interesting therapeutic candidate to treat severe malaria effects.

## 1. Introduction

Malaria is an infectious disease caused by protozoa of the genus *Plasmodium*, which includes at least five species known to infect humans: *P. falciparum*, *P. vivax*, *P. malariae*, *P. ovale*, and *P. knowlesi* [1]. It is a very severe disease that can manifest as severe anemia, with pulmonary complications that can lead to acute respiratory distress syndrome (ARDS) and cerebral malaria (CM) [2,3]. CM caused by *P. falciparum* is a neglected infectious disease, and the leading cause of morbidity and mortality in 91 endemic countries [4]. In 2021, the World Health Organization (WHO) reported more than 247 million cases of malaria, and 627 thousand deaths worldwide [4].

Animal models that mimic aspects of human malaria and allow pharmacological and therapeutic studies contribute to elucidating several questions related to *Plasmodium* spp. and humans [5]. Mice infected with *P. berghei* ANKA (PbA) simulate human CM and ARDS. Therefore, it is a murine model widely used to study the development and course of severe malaria [6].

During *Plasmodium* infection, innate immunity acts as the first line of host defense by controlling parasite growth and regulating the development of adaptive immunity and immunological memory [7]. Antigen-presenting myeloid cells, such as dendritic cells (DCs) and macrophages, recognize the parasite, leading to an immune response mediated by Toll-like receptors, amongst others [8]. DCs and macrophages produce a wide range of pro-inflammatory cytokines, including TNF, IL-12, and IL-6, as well as chemokines such as CXCR3 [9], CCL2, CXCL8, and CXCL10 [10], and CD4 T lymphocytes that mainly produce IFN-γ. These cytokines and chemokines have been associated with the susceptibility to cerebral and pulmonary malaria pathogenesis [11,12]. High levels of IFN-γ and TNF contribute to the development of CM [7,13] and pulmonary dysfunction, inducing chemokines that stimulate T lymphocytes and other leukocytes to migrate, such as Ly6C^hi^ monocytes, which contributes to severe damage to the brain and lungs [14]. In contrast, anti-inflammatory cytokines such as IL-10 control inflammatory responses, preventing tissue damage [15], and playing a protective role in experimental PbA-induced CM [16].

Medicines available for malaria include artemisinin, chloroquine (CQ), and mefloquine, which are effective drugs in combating malaria parasites and relieving patients’ pain and fever [17]. The disadvantage of these treatments is that their action is often limited to the blood phase of the disease, being ineffective in the liver phase [18,19]. Also, CQ can cause seizures in patients with epilepsy and systemic lupus erythematosus [20]. Antimalarial treatments can also cause lung dysfunction and do not guarantee protection against brain damage [21,22]. Therefore, it is urgent to identify less toxic therapeutic options against malaria.

Coumarins are common plant metabolites, and very interesting due to their pharmacological activities [23,24]. Owing to their simple and versatile structure, and chemical stability, coumarins can be easily synthesized and modified to allow more active and selective compounds [25]. When adequately substituted, they can exert various activities, including antioxidant, anti-inflammatory, and neuroprotective effects [26,27]. Changes to the scaffold make coumarins potentially efficient in treating neurodegenerative diseases [25]. Amide-functionalized coumarins have already proven to be potent molecules against these diseases, particularly in pathologies in which monoamine oxidases are involved, as well as acting as interesting neuroprotective agents [28,29,30]. Their profile as neuroprotective agents has been previously studied in neuronal cell cultures in the presence of hydrogen peroxide or lipoxygenase [28].

Our group has extensive experience in the design and synthesis of molecules with activity against neurodegenerative diseases, mainly Parkinson’s and Alzheimer’s diseases [26,28,29,30]. Some molecules have been described for their activity in in vivo models [28]. Their biological profile as neuroprotectors, able to cross the blood–brain barrier, inspired the study of these molecules in cerebral malaria in in vivo models. Based on previous studies, we hypothesized the therapeutic potential of differently substituted coumarins in treating severe experimental malaria, acting mainly as anti-inflammatory and neuroprotective agents.

From a first screening of a diverse set of synthetized coumarin-containing compounds, *N*-(coumarin-3-yl)cinnamamide (M220) stood out. After a preliminary selection of differently substituted coumarins that previously showed neuroprotective properties, by incubating the compounds with PbA-infected red blood cells and analyzing cell viability and the number of infected red blood cells, this amide-functionalized coumarin (M220) was selected for the present study as the most promising compound. After the first impressive results of the novel object recognition test, we decided to explore in depth the mechanisms involved in neuroprotection, subsequently expanding them to lung assays.

After exploring different trials, we demonstrate that M220 treatment reduces brain and lung injury, attenuating inflammation and tissue damage, and protecting mice from death. To further explore the potential of this molecule in combination with traditional antimalarial drugs, a combination with CQ (M220+CQ) has been studied. This can lead to a decrease in the CQ dose, avoiding several of its side effects. The administration of M220+CQ was shown to reduce the accumulation of microglia and leukocytes, and the production of inflammatory cytokines (TNF and IFN-γ), which are related to the upregulation of the anti-inflammatory and tissue-protective cytokine IL-10, mainly secreted by innate and adaptive immune cells. In general, the combination of M220 and CQ also promotes immunomodulatory, neuroprotective, and lung-sparing effects, being an interesting therapeutic approach to reduce the dose by combining a traditional drug with a potent neuro-lung protective agent.

## 2. Results

### 2.1. The synthesis of N-(coumarin-3-yl)cinnamamide (M220)

**Figure 1:**
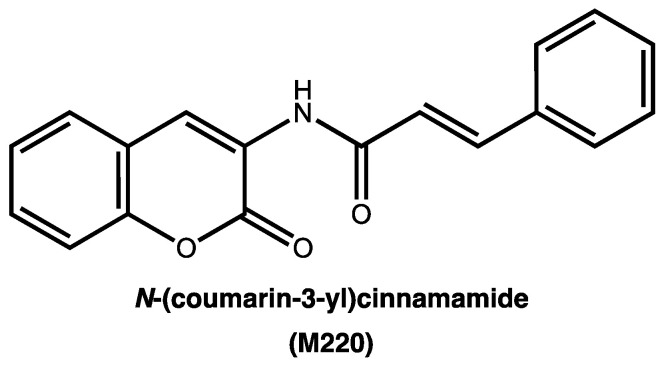
Chemical structure of *N*-(coumarin-3-yl)cinnamamide (M220).

The synthesis of M220 (Figure 1) has been performed in two different steps [31]. The first one includes the reduction of the nitro group of the commercially available 3-nitrocoumarin in hydrogen atmosphere, in the presence of ethanol and palladium catalyst, at room temperature, for five hours. After the reduction of the nitro group to the corresponding amine, the second step involves an amidation reaction using cinnamoyl chloride, pyridine, and dichloromethane, from zero degrees Celsius to room temperature, stirring overnight. After completing this protocol, the final product (M220) has been then characterized by ^1^H and ^13^C NMR and mass spectroscopy (Supplementary Figure S1).

### 2.2. Administration of M220 Protects Mice from Mortality and Preserves Memory in PbA-Infected Mice

**Figure 2:**
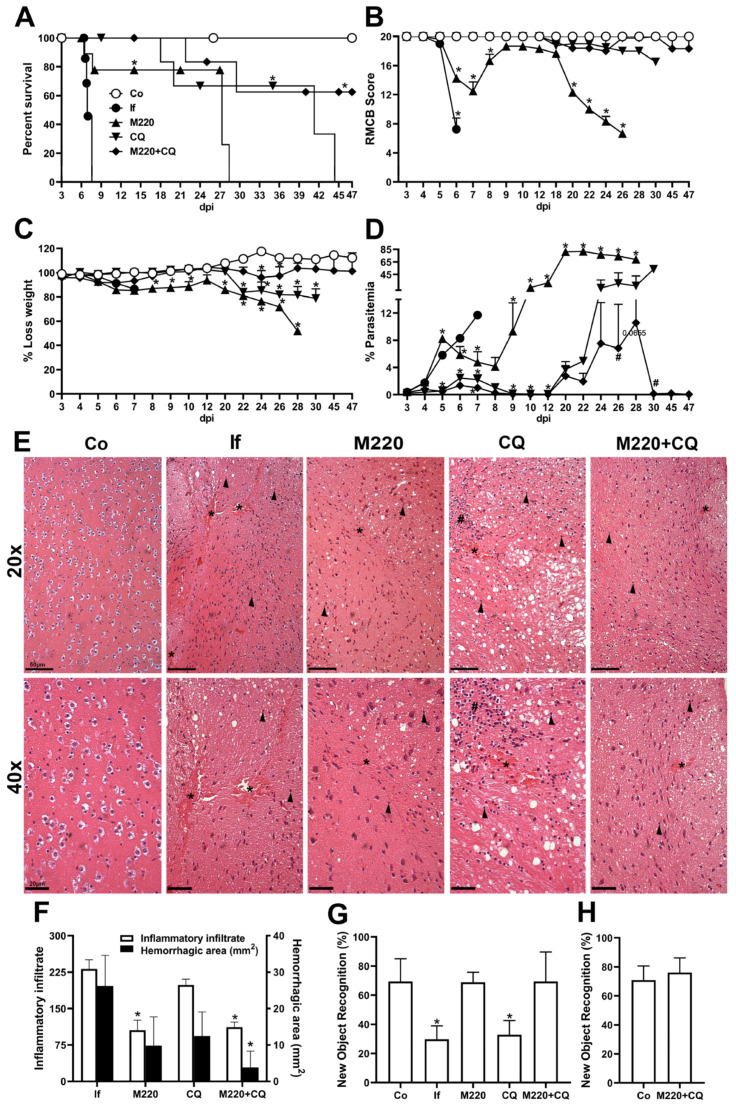
M220 increases survival and efficiently preserves cognitive functions of PbA-infected mice. C57Bl/6 mice were intraperitoneally (i.p.) infected with 1 × 10^5^ erythrocytes parasitized with PbA. Mice were treated with compound M220 (10 mg/kg) and/or CQ (30 mg/kg) via gavage, once a day, from 3 to 12 dpi. The following animal groups were created: uninfected (Co) mice that received only the vehicle; infected (If) mice that received only the vehicle; infected+M220 (M220); infected+CQ (CQ); infected+M220+CQ (M220+CQ). The following parameters have been evaluated: (**A**) survival, (**B**) clinical score using the rapid murine coma and behavior scale (RMCBS) parameter, (**C**) weight loss, (**D**) parasitemia. (**E**) Representative photomicrographs of H&E-stained brain sections (20× and 40×). Sections from an uninfected mouse with normal histological appearance (Co) and a PbA-infected mouse (If) showing multiple inflammatory infiltrates (arrow), bleeding areas (asterisks), and inflammatory foci (hashtag) at 5 dpi. In particular, treatment with M220+CQ reduced the brain injury observed at 5 dpi. The bars in the image represent 50 μm. (**F**) Hemorrhagic area (mm^2^) of brain tissue. (**G**) and (**H**) Cognitive assessment through object recognition test performed at 5 (**G**) and 47 (**H**) dpi. Data are representative of at least two independent experiments (4–6 mice/group) and shown as the mean ± SEM. Statistical analysis was performed using one-way analysis of variance (ANOVA) with Dunnett’s post-test, two-way ANOVA with Sidak’s post-test, and a log-rank (Mantel–Cox) test. # for comparison of the CQ group vs. the M220+CQ group; * for comparison of treated infected groups vs. untreated infected group. ^#^ or * *p* < 0.05.

The effects of M220 during PbA infection were investigated by analyzing general and primary parameters, such as survival, RBMC score, weight loss, and parasitemia. We chose to start mice treatment on day 3, which is the time point when mice present parasitemia, but usually no neurological damage. These findings demonstrated that mice treated with M220 had a four-fold increase in survival compared with the untreated infected group (Figure 2A). Treatment with the combination of M220 and CQ (M220+CQ) was slightly more effective, significantly increasing survival five-fold (Figure 2A). After treatments were discontinued on day 12, mice treated with M220+CQ continued to exhibit superior clinical scores compared to the scores of mice in the other treatment groups (Figure 2B). Furthermore, mice treated with M220+CQ showed reduced weight loss compared to the M220- or CQ-treated groups (Figure 2C). Figure 2D–F show the effectiveness of each treatment in controlling parasitemia, brain inflammation, and damage. M220+CQ was significantly more effective than any other treatment. It was also able to improve recrudescence after treatment discontinuation. The novel object recognition test showed that untreated and CQ-treated infected mice significantly lost their cognitive ability five days post-infection (dpi) (Figure 2G). In contrast, mice treated with M220 or M220+CQ showed full ability to recognize the novel object, similar to uninfected control mice (Figure 2G). Notably, representatives of the infected control and M220+CQ-treated groups who were still alive after 45 dpi were subjected to the new object recognition test again, demonstrating preserved cognitive ability (Figure 2H). These impressive results encouraged us to explore the mechanisms of action of M220.

### 2.3. Administration of M220 Attenuates Lung Mechanical Dysfunction during PbA Infection in Mice

**Figure 3:**
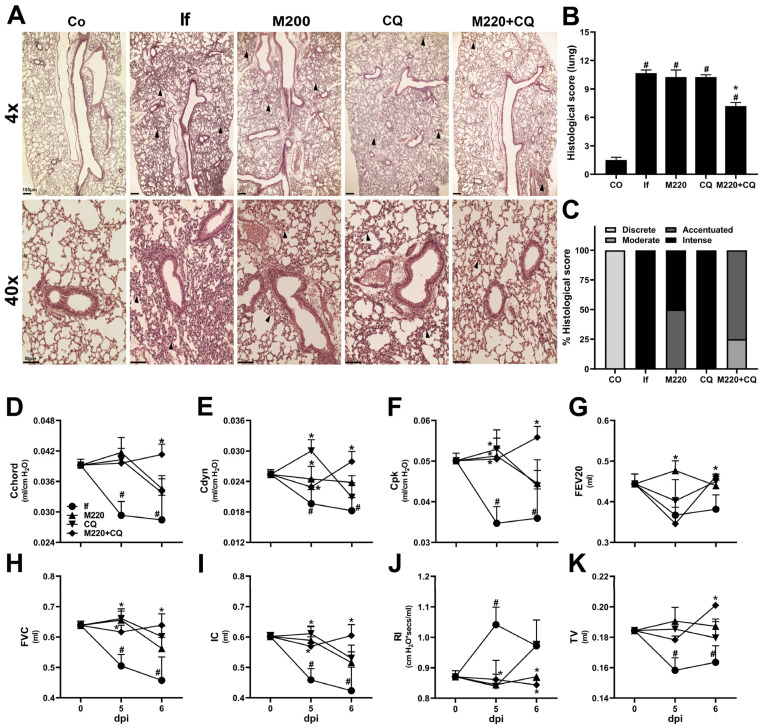
M220 improves lung mechanical capacity during PbA infection. C57Bl/6 mice were infected with PbA and treated as described in Figure 2. (**A**) Representative photomicrographs of H&E-stained lung sections (4× and 40×). Sections from an uninfected mouse with normal histological appearance (Co) and a PbA-infected mouse (If) showing thickening of the alveolar septa (arrow) at 6 dpi. Notably, treatment with M220+CQ reduced the lung injury observed at 6 dpi. The bars in the image represent 50 μm. (**B**) Histological score of lung tissue. (**C**) Descriptive analysis of 6 dpi histopathological parameters. At 5 and 6 dpi, mice were anesthetized, tracheostomized, placed in a body plethysmograph, and connected to a computer-controlled ventilator. The following parameters have been evaluated: (**D**) chord compliance (Cchord), (**E**) dynamic compliance (Cdyn), (**F**) maximum compliance (Cpk), (**G**) forced expiratory volume at 20 ms (FEV20), (**H**) forced vital capacity (FVC), (**I**) inspiratory capacity (IC), (**J**) lung resistance (Rl), and (**K**) tidal volume (TV). Data are representative of at least two independent experiments (4–6 mice/group) and are shown as the mean ± SEM. Statistical analysis was performed using one-way ANOVA variance with Tukey’s post hoc test and Student’s *t*-test. # for comparison of control groups vs. infected group; * for comparison of the CQ group vs. M220+CQ group. * or # *p* < 0.05.

Along with the brain, the lungs are the organs most affected by severe malaria. Lung dysfunction affects approximately 20% of *P. falciparum* malaria cases [32] and involves several inflammatory processes [33]. Therefore, it was hypothesized that the treatments would also decrease lung inflammation and reverse lung dysfunction seen in severe malaria. Histological evaluation revealed increased cellular infiltration and intense inflammation in untreated mice infected with PbA at 6 dpi (Figure 3A–C). In contrast, mice in the M220+CQ-treated group showed reduced histological scores and reduced inflammation (Figure 3A–C). The evaluation of lung function showed a reflection of tissue inflammation and lung injury, which led to evident mechanical dysfunction, with Rl markedly increased, a loss of all the distensibility parameters analyzed (Cchord, Cdyn, and CpK), reduced airway flow (FEV20), and low lung volumes (FVC, IC, and TV) in PbA-infected untreated mice. Treatments with M220 or CQ resulted in a modest improvement in lung function. However, treatment with M220+CQ significantly improved lung function, which normalized at 5 and 6 dpi (Figure 3D–K). Once again, the role of M220 in protecting the lungs from the side effects seen with antimalarials is very promising.

### 2.4. Effects of M220 on Brain Inflammation during PbA Infection in Mice

We next sought to determine whether modulation of brain and lung inflammation was the underlying mechanism by which compound M220 exerted its protective effect. Therefore, the cellular profiles of immune responses in these organs were evaluated. To do this, mice were infected and treated as described in Section 2.2 and Section 2.4 of the Materials and Methods, and sacrificed at 5 dpi; the brain and lungs were harvested, processed, and the recovered cells stained with specific antibodies for further flow cytometry analysis.

**Figure 4:**
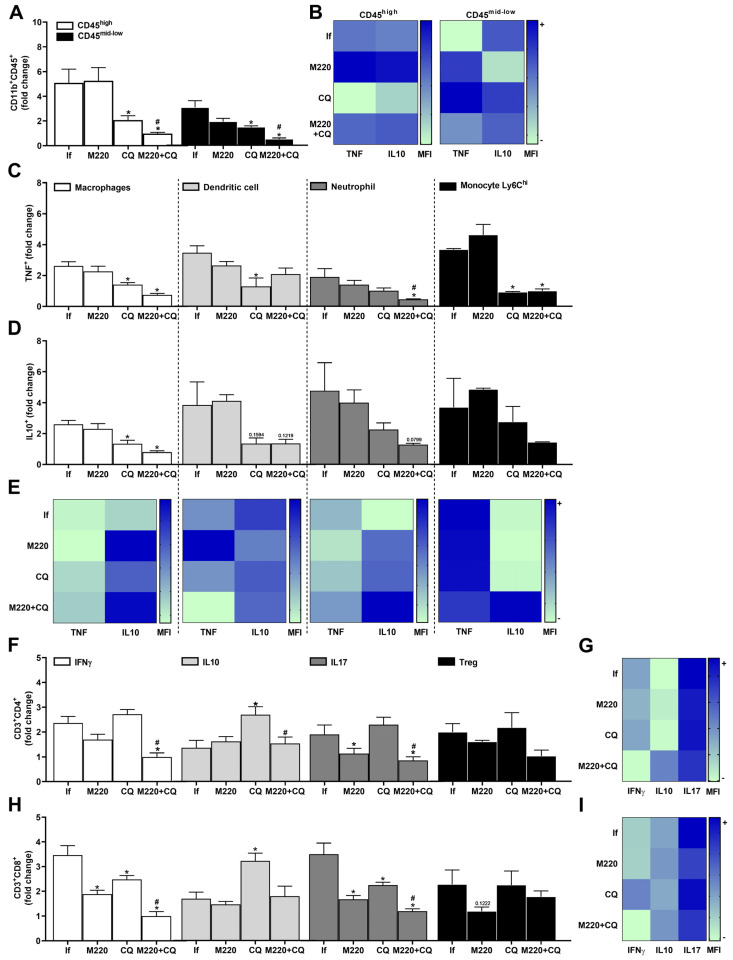
Effects of M220 on brain inflammation during PbA infection in mice. C57Bl/6 mice were infected with PbA and treated as described in Figure 2. Mice were sacrificed at 5 dpi, their brains were harvested and homogenized, and resident and sequestered cell populations were analyzed by flow cytometry. (**A**) CD11b^+^CD45^hi^ and CD11b^+^CD45^mid-low^ cells. (**B**) Heat map row representing mean fluorescence intensity (MFI) of TNF and IL-10 in CD11b^+^CD45^mid-low^; (**C**) and (**D**) CD11b^+^F4/80^+^ cells (macrophages), CD11c^+^F4/80^-^ (DCs), CD11b^+^Ly6G^+^ (neutrophils), and Ly6G^-^Ly6C^hi^ (Ly6C^hi^ monocytes) positive for TNF (**C**) and IL-10 (**D**). (**E**) Heat map representing the MFI of TNF and IL-10 in macrophages, DCs, neutrophils, and Ly6C^hi^ monocytes. (**F**) CD3^+^CD4^+^ lymphocytes were positive for IFN-γ, IL-10, IL-17A, and Foxp3 (Treg). (**G**) Heat maps representing MFI of IFN-γ, IL-10, and IL-17A in CD4^+^ lymphocytes. (**H**) CD3^+^CD8^+^ lymphocytes positive for IFN-γ, IL-10, IL-17A, and Foxp3 (Treg). (**I**) Heat map representing MFI of IFN-γ, IL-10, and IL-17A in CD8^+^ lymphocytes. In all heat maps, the color scale corresponds to relative MFI levels for each cytokine analyzed (data are presented in grayscale; black indicates the highest cytokine MFI levels, and white indicates the lowest cytokine MFI levels). Data presented in the graphs represent normalized cell number changes in uninfected control mice. Data are representative of two independent experiments (6 mice/group) and are shown as the mean ± SEM. Statistical analyses were performed using one-way ANOVA with Tukey’s post hoc test, two-way ANOVA with Sidak’s post-test, and Student’s *t*-test. # to compare the CQ group with the M220+CQ group; * for comparison of infected treated groups vs. infected untreated group. * or # *p* < 0.05.

The results showed that the number of myeloid cells (CD11b^+^CD45^hi^) and microglia (CD11b^+^CD45^mid-low^) in the brain decreased markedly with CQ treatment (Figure 4A). Notably, M220+CQ treatment was more effective than CQ, reducing both cell subsets in the brain (Figure 4A). Treatment with M220+CQ increased IL-10 production more than TNF production in both cell lines, compared to the group treated with CQ alone, which presented higher levels of TNF than IL-10 (Figure 4B). A decrease in the number of macrophages, DCs, neutrophils, and Ly6C^hi^ monocytes that produce TNF (Figure 4C) and IL-10 (Figure 4D) was found in the CQ and M220+CQ groups. The efficiency of M220+CQ to induce higher levels of IL-10 than TNF, an essential player in immunoregulation, was also observed in these leukocytes (Figure 4E). Furthermore, untreated mice infected with PbA showed robust Th1 and Th17 responses (Figure 4F), producing IFN-γ and IL-17A (Figure 4G), respectively, with lower and less activated Th2 and regulatory T cells (Tregs) (Figure 4F), producing IL-10 (Figure 4G). Moreover, a higher number of CD8^+^IFN-γ^+^ and CD8^+^IL-17A^+^ T lymphocytes was observed in the untreated infected group (Figure 4H). In contrast, PbA-infected mice treated with M220+CQ exhibited decreasing Th1 and Th17 responses (Figure 4F), followed by significantly decreased levels of IFN-γ and IL-17A, and an increase in IL-10 (Figure 4G), compared to the untreated infected group. This pattern was observed when the CD8^+^ T cells of the M220+CQ group were compared with those of the untreated infected group (Figure 4H,I).

### 2.5. Effects of M220 on PbA-Induced Inflammatory Response in the Lungs

**Figure 5:**
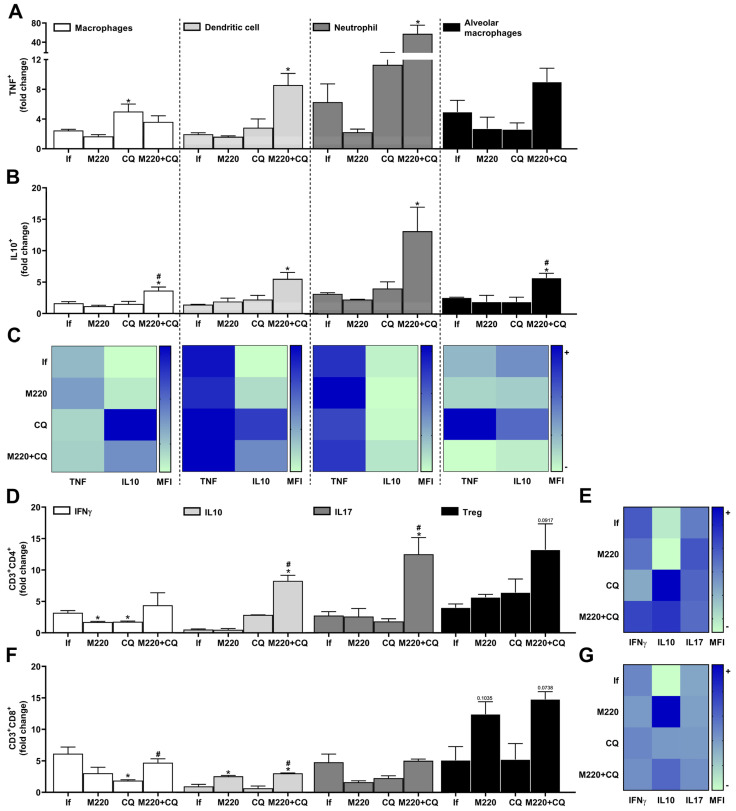
Effects of M220 on PbA-induced inflammatory response in the lung. C57Bl/6 mice were infected with PbA and treated as described in Figure 1. Mice were sacrificed at 5 dpi, lungs were harvested and homogenized, and resident and sequestered cell populations were analyzed by flow cytometry. (**A**) and (**B**) CD11b^+^F4/80^+^ (macrophages), CD11c^+^F4/80^-^ (DCs), CD11b^+^Ly6G^+^ (neutrophils), and SinglecF^+^CD11c^+^ (alveolar macrophages) cells were positive for TNF (**A**) and IL-10 (**B**). (**C**) Heat map representing the median fluorescence intensity (MFI) of TNF and IL-10 in macrophages, DCs, neutrophils, and alveolar macrophages. (**D**) CD3^+^CD4^+^ lymphocytes were positive for IFN-γ, IL-10, IL-17A, and Foxp3 (Treg). (**E**) Heat map representing the MFI of IFN-γ, IL-10, and IL-17A in CD4^+^ T cells. (**F**) CD3^+^CD8^+^ lymphocytes were positive for IFN-γ, IL-10, IL-17A, and Foxp3 (Treg). (**G**) Heat map representing the MFI of IFN-γ, IL-10, and IL-17A in CD8^+^ T cells. In all heat maps, the color scale corresponds to relative levels of the MFI for each cytokine analyzed (data are presented in grayscale; black indicates the highest cytokine MFI levels, and white indicates the lowest cytokine MFI levels). The data presented in the graphs represent fold-changes in cell numbers normalized to uninfected control mice. Data are representative of at least two independent experiments (three mice/group) and shown as the mean ± SEM. Statistical analysis was performed using one-way ANOVA variance with Tukey’s post hoc test and Student’s *t*-test. # for comparison of CQ group vs. M220+CQ group; * for comparison of infected treated groups vs. infected untreated group. * or # *p* < 0.05.

PbA infection induced a marked increase in the number of cytokine-releasing cells in the lung: three-fold for TNF- and DC-producing macrophages, and five-to-seven-fold for TNF-producing neutrophils and alveolar macrophages (Figure 5A). Furthermore, although in lower quantities than TNF^+^ cells, a greater number of IL-10-producing macrophages, DCs, neutrophils, and alveolar macrophages were found in the lungs after PbA infection (Figure 5B). Although CQ treatment increased the number of TNF-producing macrophages (Figure 5A), it reduced the levels of TNF and increased the levels of IL-10 production by these cells during infection **(**Figure 5C). Treatment with the M220+CQ increased the number of TNF^+^ DCs and neutrophils (Figure 5A) but reduced the levels of TNF production by alveolar macrophages (Figure 5C). Notably, this treatment induced a greater number of macrophages, DCs, neutrophils, and alveolar macrophages positive for IL-10 (Figure 5B). In addition, treatment with the M220+CQ stimulated these cells, except alveolar macrophages, to produce higher levels of this anti-inflammatory cytokine, in comparison with infected untreated mice (Figure 5C). The results also demonstrated that M220+CQ affected the profile of Th cells during infection by increasing IL-10- and IL-17A-positive cells and Treg lymphocytes (Figure 5D). Moreover, M220+CQ treatment resulted in higher levels of IL-10 production than IFN-γ and IL-17A production by CD4^+^ and CD8^+^ T lymphocytes (Figure 5E,G). Notably, M220 treatment potentiated IL-10 production by Treg CD8^+^ cells (Figure 5G). These data corroborate the potential for M220 to act as an anti-inflammatory agent during severe malaria progression.

### 2.6. Effects of M220 on Innate and Adaptive Immune Cell Populations in the Spleen of PbA-Infected Mice

**Figure 6:**
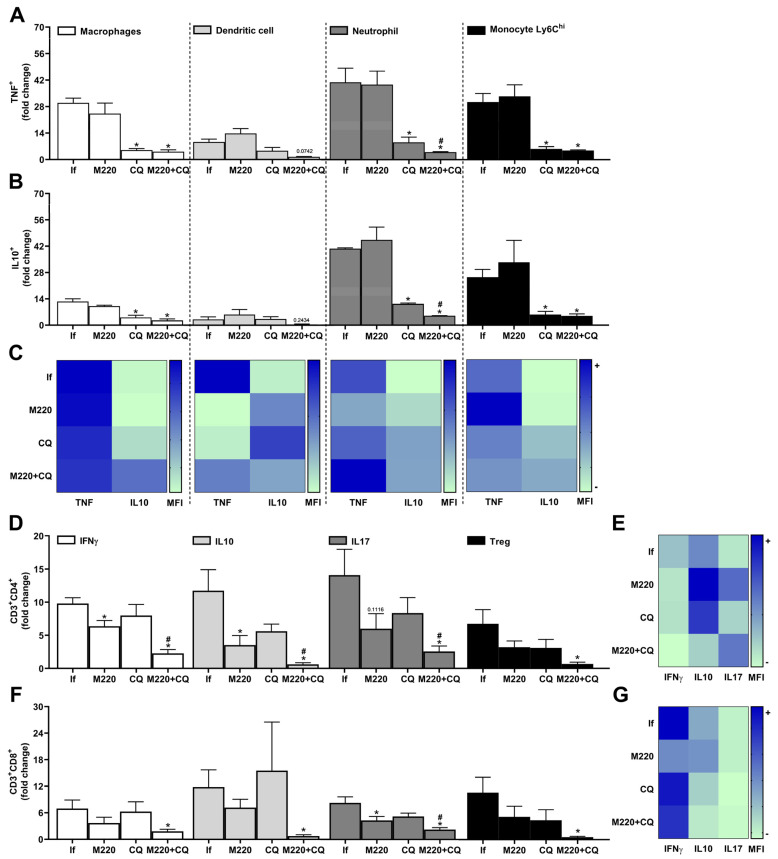
Effects of M220 on innate and adaptive cells profile in the spleen of PbA-infected mice. C57Bl/6 mice were infected with PbA and treated as outlined in the legend of Figure 2. Mice were sacrificed at 5 dpi, spleens were harvested and homogenized, and splenocytes were analyzed by flow cytometry. (**A**) and (**B**) CD11b^+^F4/80^+^ cells (macrophages), CD11c^+^F4/80^-^ (DCs), CD11b^+^Ly6G^+^ (neutrophils), and Ly6G^-^Ly6C^hi^ (Ly6C^h^ monocytes) were positive for TNF (**A**) and IL-10 (**B**). (**C**) Heat map depicting the mean fluorescence intensity (MFI) of TNF and IL-10 in macrophages, DCs, neutrophils, and Ly6C^h^ monocytes. (**D**) CD3^+^CD4^+^ lymphocytes were positive for IFN-γ, IL-10, IL-17A, and Foxp3 (Treg). (**E**) Heat map representing the MFI of IFN-γ, IL-10, and IL-17A in CD4 + T cells. (**F**) CD3^+^CD8^+^ lymphocytes were positive for IFN-γ, IL-10, IL-17A, and Foxp3 (Treg). (**G**) Heat map representing the MFI of IFN-γ, IL-10, and IL-17A in CD8^+^ T cells. In all heat maps, the color scale corresponds to the relative levels of MFI for each cytokine analyzed (data are presented in grayscale; black indicates the highest cytokine MFI levels, and white indicates the lowest cytokine MFI levels). Data presented in the graphs represent normalized cell number changes in uninfected control mice. Data are representative of two independent experiments (six mice/group) and are shown as the mean ± SEM. Statistical analysis was performed using one-way variance ANOVA with Tukey’s post hoc test and Student’s *t*-test. # to compare the CQ group with the M220+CQ group; * for comparison of infected treated groups vs. infected untreated group. * or # *p* < 0.05.

The spleen contains several immune and stromal cells, providing an environment for tracking the blood in search of foreign molecules and organisms, and activating immune cells [34]. During PbA infection, infected red blood cells circulate through the body, the spleen being an essential organ for the activation and/or generation/expansion of immune cells against this infection. Treatment with M220+CQ reduced the number of TNF^+^ and IL-10^+^ macrophages, DCs, neutrophils, and Ly6C^hi^ monocytes (Figure 6A,B) compared to the infected untreated group. Notably, treatment with M220+CQ affected the stability of TNF and IL-10 cytokine production by reducing TNF and augmenting IL-10 levels by these cell subsets, except for neutrophils (Figure 6C). Moreover, M220+CQ-treated mice suffered a profound reduction in Th1, Th2, Th17, and Treg lymphocytes (Figure 6D), and also reduction in CD8^+^ T cells producing IFN-γ, IL-10, or IL-17A, and CD8^+^ Treg lymphocytes (Figure 6F). In the spleen, treatment with M220+CQ, but not CQ alone, reduced the production of IFN-γ and IL-10 by CD4^+^ and CD8^+^ T lymphocytes when compared with infected untreated mice (Figure 6E,G).

## 3. Discussion

Severe malaria has a high mortality rate. This disease produces intense inflammation, causing several health complications [35]. In addition to death, the main challenge for malaria infection is the side effects of antimalarial drugs, which can cause symptoms to persist or even worsen after treatment [36,37]. The treatment proposed here with compound M220 prolonged the survival of mice four-fold. The combination of M220 and CQ increased survival more than six-fold, compared to untreated infected mice. M220+CQ guarantees approximately 60% of mice survival, which is much higher than the results obtained with CQ alone.

Clinical signs of weakness, pain, and apparent exhaustion have been described in human malaria [38]. PbA infection provides some of these clinical signs that mimic the clinical condition [39,40]. Importantly, treatment with M220 significantly improved discomfort and clinical score, suggesting that this has promise in alleviating suffering caused by infection. Human malaria also causes nausea and loss of appetite, often making it difficult for patients to eat properly, leading to weight loss [41]. When comparing treatments, M220+CQ, but not CQ alone, prevented weight loss at all time points, suggesting that these mice maintained their appetite and food intake at an average level. Once again, M220 avoids some of the drawbacks of using CQ.

Severe malaria can be fatal if not treated quickly [4], and even with effective treatments, it can cause serious harm to affected humans [17,20]. The severe malaria model used in this study can lead to the development of cerebral malaria in mice, allowing better exploration of ways to address the potential adverse effects caused by current therapies and the sequelae that cannot be avoided, such as loss of cognitive ability [39]. CQ controls parasite growth, but has specific side effects [17], and does not avoid the central nervous system (CNS) harm effects [17,42]. The data presented here confirm the literature [17,43], showing that the PbA infection process leads to loss of the ability to form memories. This ability was lost in CQ-treated mice. Notably, treatments with M220 alone or M220+CQ preserved the mice’s ability to form memories, similar to those obtained in uninfected mice. This neuroprotection turned out to be permanent, considering that the treatment was interrupted after 12 dpi, and the mice retained their ability to form memories at least until 47 dpi, the last time point evaluated. This indicates that M220 is acting efficiently in the mice brain, presenting a substantial and persistent neuroprotective effect.

The balance between pro- and anti-inflammatory responses determines the outcome of infection [41]. Microglial cells are found in the CNS parenchyma and share several characteristics with macrophages [44]. After neuronal or tissue damage, microglial cells change their shape and immunocytochemical phenotype to transition to the activated state [43,45]. Microglial function is important against infectious agents by participating in the initial immune response and the recruitment of peripheral cells of the immune system, such as neutrophils, monocytes, and T lymphocytes, to the site of infection [44,46]. The number of microglia and migrating cells increased in PbA-infected brain tissue. In addition, treatment with M220+CQ decreased the number of microglia and migrating cells, reaching levels similar to those found in the brains of uninfected mice.

Pro-inflammatory responses, including the production of TNF and IFN-γ, are essential for protection against pathogens by presenting an antiparasitic effect [47]. However, the association of TNF and IFN-γ with other pro-inflammatory cytokines is known to cause severe malaria [6,47]. Treatment with M220+CQ significantly decreased the number of macrophages, DCs, neutrophils, and Ly6C^hi^ monocytes producing TNF in the brain during PbA infection. This treatment also reduced the number of TNF-positive Ly6C^hi^ monocytes and levels of these cytokines produced by them in the brain. Ly6C^hi^ monocytes are an extremely important cellular subtype associated with the severity of cerebral malaria [48]. When they accumulate in the brain, they can be lethal and/or aggravate neurological manifestations [49,50].

In addition to TNF, the cytokines IFN-γ and IL-17A have also been associated with an increased risk of developing cerebral malaria [51]. Although IFN-γ can induce protective immune responses against parasitemia, reinfection, and anemia [52], its reduction or absence significantly decreases malaria pathogenesis [12,53]. Treatment with M220+CQ reduced the number of CD4^+^ and CD8^+^ T cells positive for IFN-γ and IL-17A and decreased the production of these cytokines. As IFN-γ is also associated with worse cognitive tasks involving the hippocampus, and the absence of IFN-γ leads to better cognitive performance [54], a mechanism by which treatment with M220 protects memory is modulation of the production of key cytokines involved in malaria pathogenesis. Another important finding that may be associated with the described protection was the increase in the number of IL-10-positive CD4^+^ and CD8^+^ T lymphocytes. It is well-known that IL-10 plays a regulatory role in the development of pathogenesis associated with severe malaria, inducing an anti-inflammatory environment [16]. Importantly, the M220+CQ treatment also increased the levels of IL-10 production by macrophages, neutrophils, and microglia in the brain during PbA infection. Therefore, M220+CQ treatment decreased the number of resident and recruited cells of innate and adaptive immune responses, producing pro-inflammatory cytokines and promoting a less inflammatory environment in the brain.

Severe malaria may also affect the lung [3,55,56], inducing malaria-associated acute respiratory distress syndrome, a complication caused by the disease despite treatment with antimalarial drugs [3,12]. In the current study, PbA-infected untreated mice had impaired lung capacity. Treatment with M220+CQ demonstrated better effects in maintaining lung function than the other groups. These findings demonstrate an additional effect of compound M220 in treating severe malaria and protecting lung capacity/function in addition to CNS/cognitive activity. Lung protection may be related to increased numbers of resident (alveolar macrophages) and migrated (macrophages, DCs, and neutrophils) IL-10-positive cells, as well as increased levels of IL-10 production by these cells.

Alveolar macrophages are the main source of IL-10 in healthy lungs and are constitutively secreted under normal conditions [56]. Studies in mice have identified an anti-inflammatory role for IL-10 as a preventive agent against potential tissue damage due to the inflammation [16,56]. These findings corroborate our hypothesis that increased IL-10 production is the explanation for why the lungs of mice treated with M220 exhibit characteristics of non-fibrotic lungs. Furthermore, a significant reduction in IFNγ was observed in the lung and, as has been mentioned for the brain, a favorable scenario is drawn to improve protection against the development of the pathogenesis of the disease. Additionally, another major benefit of M220 was the increased number of Tregs, both CD4^+^ and CD8^+^, despite the increase in Th17. The increase in Th2 cells and Tregs can counteract the presence of Th17, thus protecting against lung injury. Notably, IL-17A may be detrimental to lung health in cases of acute lung injury [57,58], although this cytokine protects against various pathogens. For example, IL-17A-deficient mice are more susceptible to different respiratory pathogens, including fungi [59] and bacteria [60]. From our results, we can infer that during malaria infection, Th17 cells do not show their harmful characteristics during treatment. Further research is needed to understand how it positively helps protect treated mice.

The spleen is a crucial organ for the clearance of parasitized red blood cells and the generation of immunity during malaria [61]. It is also the primary site where the adaptive immune response against the blood stage of *Plasmodium* infection is initiated [62]. A lower number of innate immune cells were found in the spleens of of mice treated with CQ and M220+CQ. Effective CQ-combating of the parasite at the blood stage [19] may contribute to reducing the generation/differentiation of macrophages, DCs, neutrophils, and Ly6C^hi^ monocytes and their migration into the spleen. However, treatment with M220+CQ, but not CQ alone, decreased the number of all lymphocyte subsets tested, compared to that of untreated infected mice. Notably, the reduced cells that still migrated and/or were generated in the spleen maintained a prevalent anti-inflammatory environment, producing lower TNF and higher IL-10 levels (macrophages, DCs, and Ly6C^hi^ monocytes) and lower IFN-γ levels (CD4^+^ and CD8^+^ lymphocytes), compared to untreated infected mice.

The chemokine milieu induced during M220+CQ treatment may also have contributed to CNS and lung protection, reducing migration and/or expansion of the inflammation. However, future analysis is necessary to confirm or investigate this hypothesis, mainly by evaluating the production levels of CCL2, CCL4, CXCL4, CXCL8, CXCL10, and the receptors CXCR3 and CCR2, correlated with susceptibility to cerebral malaria and lung inflammation [9,10,11]. As future perspectives, our research group aims to investigate the effects of the compound M220 (i) when combined with other antimalarial drugs commonly prescribed in clinic settings; and (ii) on various parasite strains, particularly those that have shown resistance to different antimalarial drugs. Furthermore, M220 may be a promising candidate to be studied against different pathologies in which infectious and inflammatory processes are involved.

## 4. Materials and Methods

### 4.1. Synthesis of N-(coumarin-3-yl)cinnamamide (M220)

*General remarks.* Starting materials and reagents were obtained from commercial suppliers (Sigma-Aldrich, St. Louis, MO, USA) and were used without further purification. Melting points (Mp) are uncorrected and were determined with a Reichert Kofler thermopan or in capillary tubes in a Büchi 510 apparatus. ^1^H NMR (300 MHz) and ^13^C NMR and DEPT (75.4 MHz) spectra were recorded with a Bruker AMX spectrometer using CDCl_3_ as solvent. Chemical shifts (*δ*) are expressed in parts-per-million (ppm) using TMS as an internal standard. Coupling constants *J* are expressed in Hertz (Hz). Spin multiplicities are given as s (singlet), d (doublet), and m (multiplet). Mass spectrometry was carried out with a Hewlett-Packard 5988A spectrometer. Flash chromatography (FC) was performed on silica gel (Merck 60, 230–400 mesh); analytical TLC was performed on precoated silica gel plates (Merck 60 F254). Organic solutions were dried over anhydrous sodium sulfate. Concentration and evaporation of the solvent after reaction or extraction was carried out on a rotary evaporator (Büchi Rotavapor) operating under reduced pressure. The analytical results document ≥ 97% purity for the final compound M220.

*Procedure for the preparation of the 3-aminocoumarin.* The commercially available 3-nitrocoumarin (1.0 mmol) was dissolved in ethanol (10 mL). Palladium on carbon (Pd/C, catalytic amount) was added, and the suspension stirred in hydrogen gas (H_2_) atmosphere for 5 h. The batch was evaporated and purified by flash chromatography, using hexane/ethyl acetate (9:1), to give the 3-aminocoumarin in 95% yield.

*Procedure for the preparation of the N-(coumarin-3-yl)cinnamamide (M220)*. To a mixture of 3-aminocoumarin (1.0 mmol) and pyridine (1.1 mmol) in dichloromethane (5 mL), cinnamoyl chloride (1.1 mmol) was added dropwise at 0 °C, with constant stirring. The reaction mixture was stirred at 0 °C to room temperature overnight. The organic phase was then partitioned at first with saturated NaHCO_3_, and then with water. The organic portions were combined and dried over anhydrous Na_2_SO_4_ and filtered, and the solvent was evaporated to give a crude product. It was then purified by flash chromatography, using hexane/ethyl acetate (9:1), to give *N*-(coumarin-3-yl)cinnamamide (M220, 59%). M.p.: 215 °C. ^1^H NMR (CDCl_3_) δ (ppm), *J* (Hz): 6.68 (d, 1H, *J* = 15.6, CH), 7.31–7.63 (m, 9H, ArH), 7.81 (d, 1H, *J* = 15.6, CH), 8.34 (s, 1H, NH), and 8.88 (s, 1H, ArH). ^13^C NMR (CDCl_3_) δ (ppm): 116.6, 120.3, 122.5, 124.4, 125.5, 125.7, 128.6, 129.7, 130.4, 130.7, 135.4, 141.9, 150.4, 158.2, and 165.8. DEPT (CDCl_3_) δ (ppm): 116.6, 122.5, 124.4, 125.7, 128.6, 129.7, 130.4, 130.7, and 141.9. EI-MS m/z (%): 291.

### 4.2. Ethics Statement

C57Bl/6 female mice (aged 8–10 weeks) were obtained from the Central Animal Hospital (ICB—UFMG). The mice were maintained in microisolators containing food and water ad libitum, according to the Brazilian Guidelines on Animal Work and the Guide for the Care and Use of Laboratory Mice of the National Institutes of Health (NIH). This study was approved by the Animal Ethics Committee (CEUA) of the Universidade Federal de Minas Gerais (permit number: 345/2023).

### 4.3. Infection

The mice were infected with the *Plasmodium berghei* ANKA (PbA) green fluorescent protein (GFP) clone cl15cy1 strain (which constitutively expresses GFP throughout the life cycle), donated by Prof. Cláudio Romero Farias Marinho (Departamento de Parasitologia, Universidade de São Paulo, USP). Mice were infected intraperitoneally (i.p.), with a standardized inoculum of 1 × 10^5^ parasitized red blood cells (pRBCs) per mouse in sterile phosphate buffer solution (PBS, 200 µL). Parasitemia was determined through analysis of GFP expression by flow cytometry, as previously described [6,37]. Briefly, a drop of tail whole blood was collected from mice infected or not with PbA. The blood was directly placed into a polystyrene tube containing 2 mL of sterile PBS for flow cytometry analysis using FACS Canto II (Becton Dickinson, San José, CA, USA). The GFP frequency was measured using a laser (488 nm), and the data were analyzed using FlowJo software (version 10). The erythrocyte population was identified on their morphological characteristics in dot plot graphic (FSCxSSC), and then was analyzed for the presence of GFP^+^. A total of 100,000 events were acquired for each sample. The mice were observed daily throughout the infection period for parasitemia, weight loss, and survival. The clinical signs of CM were assessed daily using a rapid murine coma and behavior scale [63].

### 4.4. Treatments

The mice were infected as described above. After 3 days post-infection (dpi), when parasitemia became detectable in the bloodstream, they were treated orally by gavage with **M220** (10 mg/kg dose administered for in vivo treatments in mice, and similarly a safe dose of selective MAO-B inhibitors [28], without any observed toxic effects) and/or CQ (30 mg/kg) once daily for 10 days, diluted in 0.5% carboxymethyl cellulose (vehicle).

### 4.5. Novel Object Recognition Test

The test was conducted in an open field measuring 40 × 60 cm, delimited by four walls 50 cm in height [6]. On the first day (3 dpi), habituation was performed. The animal was carefully placed in the rear left corner of the device, and the environment was explored for 5 min. On the second day (4 dpi), the animal was returned to the device, in which there were two objects of the same shape, size, and color (A1 and A2) for an exploration time of 10 min. The next day (24 h later) (5 dpi), long-term memory and response to the placement of a new object (B), in an exploration time of 5 min, were evaluated. Notably, representatives of the control and M220+CQ-treated infected groups still alive after 45 dpi were subjected again to the new object recognition test. Briefly, exploratory preference was defined as the percentage of total exploration time spent investigating a familiar object (A) or new object (B), calculated for each animal:

TB/(TA + TB) × 100

where TA = time spent exploring familiar object A and TB = time spent exploring new object B. The distance covered in the arena of the apparatus was recorded as a parameter of locomotor activity. Anymaze software (https://www.any-maze.com) (Stoelting Co., Wood Dale, IL, USA) was used for the behavioral analysis.

### 4.6. Spirometry

Mice were infected with 1 × 10^5^ pRBCs, treated or not with M220 and/or CQ, anesthetized with an association of xylazine (12.5 mg/kg, Syntec do Brasil Ltd.a, Barueri, Brazil) and ketamine (100 mg/kg, Syntec do Brasil Ltd.a) subcutaneously, and subjected to invasive spirometry (Buxco Research Systems, Wilmington, NC, USA) [64]. Under mechanical respiration, the tidal volume (TV), dynamic compliance (Cdyn), and lung resistance (Rl) were determined using resistance and compliance (RC) tests. To measure chord compliance (Cchord) and peak of compliance (Cpk), the lungs were inflated to a standard pressure of +30 cm H_2_O and then slowly exhaled until a negative pressure of −30 cm H_2_O was reached. Cchord was evaluated at +10 cm H_2_O and Cpk was determined by the pressure/volume ratio at the peak. Forced vital capacity (FVC) and inspiratory capacity (IC) were recorded during this maneuver. The fast-flow volume maneuver was performed, and the lungs were first inflated to +30 cm H_2_O and immediately subjected to a highly negative pressure to enforce expiration until −30 cm H_2_O, and the forced expiratory volume at 20 ms (FEV20) was recorded.

### 4.7. Immunophenotyping by Flow Cytometry 

Mice infected with PbA were euthanized at 5 dpi. The brains and spleens were removed and processed according to the methodology described by Brant et al. [37]. Lungs were removed and processed according to the methodology described by Claser et al. [12]. Purified cells from the brain, lung, and spleen were plated and incubated with brefeldin A (10 μg/mL) (Invitrogen, Waltham, MA, USA) for 3 h at 37 °C in the presence of 5% CO_2_. The cells were blocked with Fc Block (antibody CD16/CD32 in PBS/BSA 1%), followed by staining with specific combinations of antibodies for cell surface molecule labeling: CD3 and CD11b (APC-Cy7); CD4 and Ly6C (PE-Cy7); CD8, Ly6G, and SinglecF (BV421); CD25 and CD45 (PerCP-Cy5.5); F4/80 (FITC); CD11c (V500); and isotype controls (all antibodies from BD Biosciences, Franklin Lakes, NJ, USA). For intracellular staining, the following antibodies were added: IFN-γ (Alexa 488), IL-17A, Foxp3 (PE), TNF (PE), and IL-10 (APC). A total of 30,000 cells (events) were acquired using a FACS Canto II cytometer (BD Biosciences) and analyzed using FlowJo software (ver. 10). Our gating strategy is shown schematically in Supplementary Figure S1 (for innate immune cells subset composition) and Supplementary Figure S2 (for adaptative immune cells subset composition).

### 4.8. Histopathological and Morphometric Analysis

After mechanical procedures and bronchoalveolar lavage, mice were euthanized. The lungs and brain were removed and immediately fixed in 4% buffered formalin for 72 h. Then, the samples were gradually dehydrated in ethanol, diaphanized in xylol, and embedded in paraffin. Next, 5 μm thick sections were cut with which histopathological slides were prepared and stained with hematoxylin and eosin (H&E) for histopathological, semiquantitative, and morphometric analyses. All histopathological analyses were performed blindly. The lungs were analyzed to assess inflammation in the airway and perivascular and parenchymal regions. The score was based on a previously described method [65]. For histopathological analysis, the brain fragments were analyzed for the presence of congestion, hemorrhage areas, and cellular infiltration. The morphometric analysis was performed by adapting the methodology previously described [66]. To quantify the inflammatory infiltrate, 20 randomized images of the cerebral cortex at 40× magnification were digitized, and the infiltrate was identified and manually quantified using a cursor.

### 4.9. Statistical Analysis

Statistical significance was assessed using GraphPad Prism 8.0 (GraphPad Software, San Diego, CA, USA). Student’s *t*-test, one-way analysis of variance (ANOVA), Tukey’s multiple comparisons post-test, two-way ANOVA, and Sidak’s multiple comparisons post-test were used as described in each figure legend. Data are represented as mean ± SEM, with significance set at *p* ≤ 0.05. Statistical analyses were performed using GraphPad Prism 8.0.1 (GraphPad Software).

## 5. Conclusions

After a preliminary screening of a series of coumarins with neuroprotective properties, *N*-(coumarin-3-yl)cinnamamide (M220) has been synthesized and explored for its in vivo activity against severe malaria. After analyzing the first PbA model results, showing impressive cognitive insights, a study on the anti-inflammatory and neuroprotective properties, based on an extensive immunological profile, has been performed. Overall, the present study suggests that compound M220, mainly combined with CQ, exerts immunomodulatory, neuroprotective, and pulmonary function-preserving effects. The treatment proved to be effective on both organs mainly affected by severe malaria: brain and lungs. Moreover, M220 proved to be ideal for oral administration in the mice model, making it a very interesting therapeutic option for the treatment of this severe disease.

## Data Availability

Data will be made available on request.

## Supplementary Materials

The following supporting information can be downloaded at: https://www.mdpi.com/article/10.3390/ph17010046/s1, Figure S1: (A) Synthetic methodology and reaction conditions for obtaining *N*-(coumarin-3-yl)cinnamamide (M220). The synthesis was performed in two steps, as described previously. (B) Proton nuclear magnetic resonance (^1^H NMR) spectrum. ^1^H NMR spectroscopy was used to determine the chemical structure. The presence of a broad singlet at 8.34 ppm was compatible with the nitrogen proton of the amide group. In addition, the presence of a duplet at 6.68 ppm, with a coupling constant of 15.6 Hz, is compatible with the hydrogen of the double bond contiguous to the carbonyl group of the amide (a *trans* isomer). (C) Carbon nuclear magnetic resonance (^13^C NMR) spectrum, with the DEPT spectrum in the black box. (D) Electron ionization (EI) mass spectrum. (E) High performance liquid chromatography (HPLC) trace and purity index; Figure S2: A schematic representation of gating strategy for innate immune subpopulations isolated from spleen, lung and brain of mice and stained for IL-10 and TNF cytokine production, and corresponding dotplots of IL-10^+^ macrophages (and/or alveolar macrophages and/or microglia), neutrophils, dendritic, and TNF^+^ macrophages (and/or alveolar macrophages and/or microglia), neutrophils and dendritic cells; Figure S3: A schematic representation of gating strategy for adaptative immune cells subpopulations isolated from spleen, lung and brain of mice and stained for IL-10, IFN-g and IL-17 cytokine production, and corresponding dotplots of: IL-10^+^CD4^+^ T cells, IFN-g^+^CD4^+^ T cells, IL-17^+^CD4^+^ T cells; IL-10^+^CD8^+^ T cells, IFN-g^+^CD8^+^ T cells, IL-17^+^CD8^+^ T cells; and IL-10^+^CD4^+^Treg cells and IL-10^+^CD8^+^Treg cells.
