# Supplementary material for: N-(coumarin-3-yl)cinnamamide Promotes Immunomodulatory, Neuroprotective, and Lung Function-Preserving Effects during Severe Malaria
Source: Pharmaceuticals (Basel). 2023 Dec 27;17(1):46. doi: 10.3390/ph17010046 (PMC10821074; doi:10.3390/ph17010046)
Supplement: Supplementary file 1 [file pharmaceuticals-17-00046-s001.zip › Supplementary Figure S1.pdf]

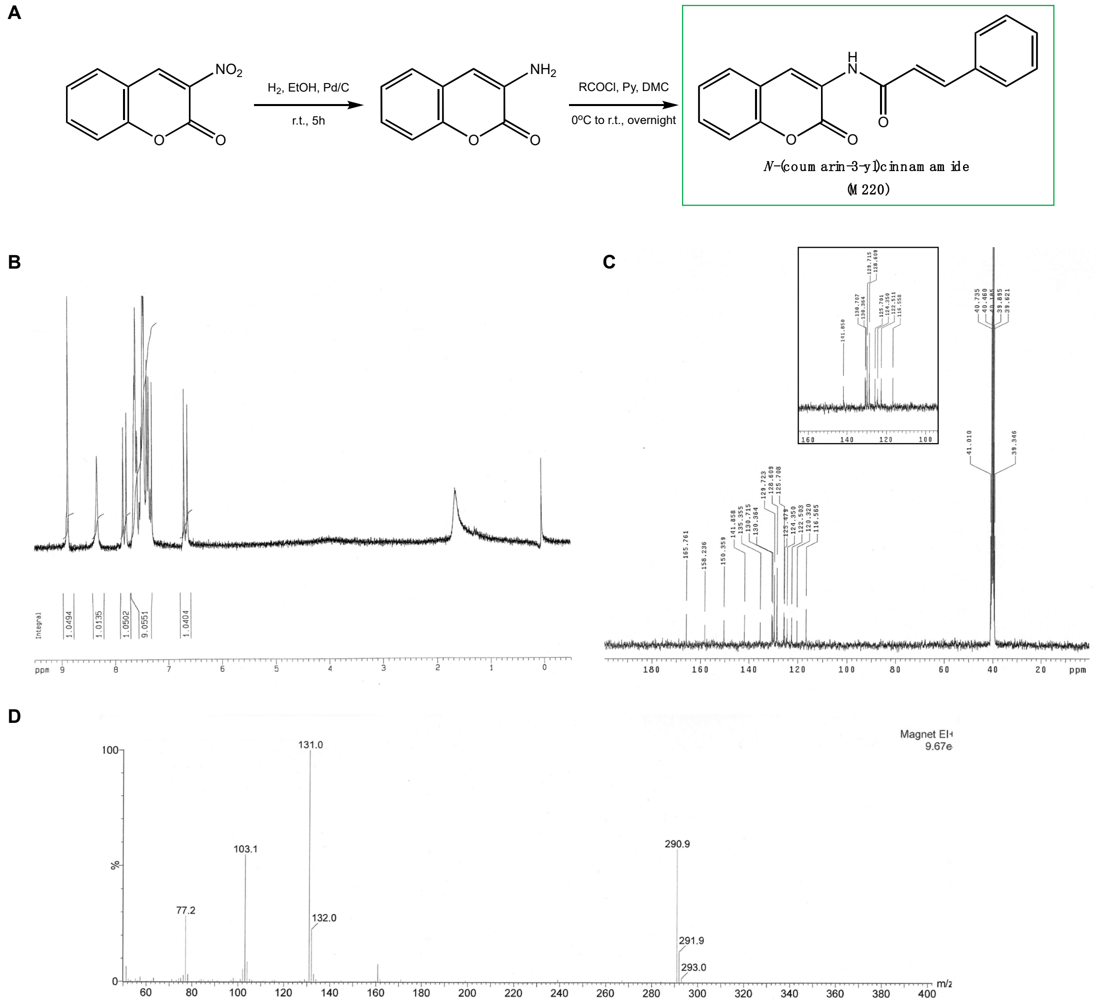

E

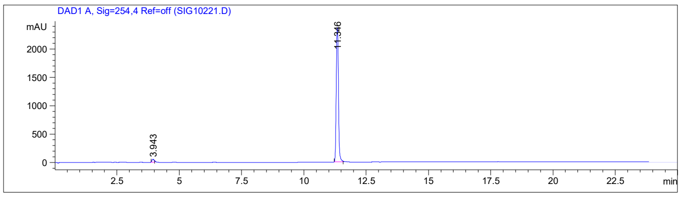

Peak #	RetTime [min]	Type	Width [min]	Area [mAU*s]	Height [mAU]	Area %
1	3.943	MM	0.0984	317.91272	53.86581	2.1702
2	11.346	MM	0.1010	1.43314e4	2364.34082	97.8298

Supplementary Figure S1. (A) Synthetic methodology and reaction conditions for obtaining *N*-(coumarin-3-yl)cinnamamide (**M220**). The synthesis was performed in two steps, as described previously. **(B)** Proton nuclear magnetic resonance (^{1}H NMR) spectrum. ^{1}H NMR spectroscopy was used to determine the chemical structure. The presence of a broad singlet at 8.34 ppm was compatible with the nitrogen proton of the amide group. In addition, the presence of a duplet at 6.68 ppm, with a coupling constant of 15.6 Hz, is compatible with the hydrogen of the double bond contiguous to the carbonyl group of the amide (a *trans* isomer). **(C)** Carbon nuclear magnetic resonance (^{13}C NMR) spectrum, with the DEPT spectrum in the black box. **(D)** Electron ionization (EI) mass spectrum. **(E)** High performance liquid chromatography (HPLC) trace and purity index.
