# Supplementary material for: N-(coumarin-3-yl)cinnamamide Promotes Immunomodulatory, Neuroprotective, and Lung Function-Preserving Effects during Severe Malaria
Source: Pharmaceuticals (Basel). 2023 Dec 27;17(1):46. doi: 10.3390/ph17010046 (PMC10821074; doi:10.3390/ph17010046)
Supplement: Supplementary file 1 [file pharmaceuticals-17-00046-s001.zip › Supplementary Figure S2.pdf]

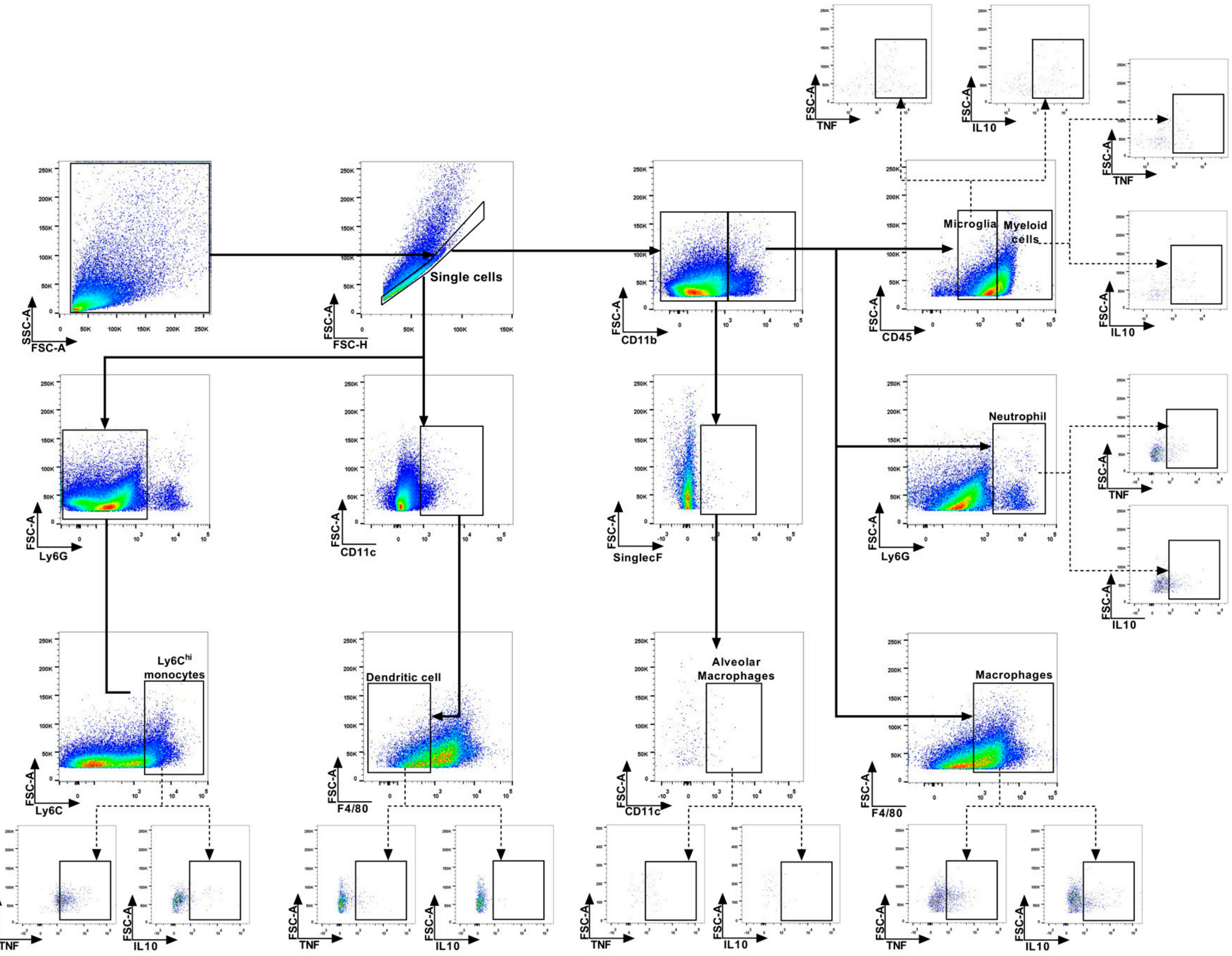

Supplementary Figure S2. A schematic representation of gating strategy for innate immune subpopulations isolated from spleen, lung and brain of mice and stained for IL-10 and TNF cytokine production, and corresponding dotplots of IL-10^{+} macrophages (and/or alveolar macrophages and/or microglia), neutrophils, dendritic, and TNF^{+} macrophages (and/or alveolar macrophages and/or microglia), neutrophils and dendritic cells.
