# Supplementary material for: N-(coumarin-3-yl)cinnamamide Promotes Immunomodulatory, Neuroprotective, and Lung Function-Preserving Effects during Severe Malaria
Source: Pharmaceuticals (Basel). 2023 Dec 27;17(1):46. doi: 10.3390/ph17010046 (PMC10821074; doi:10.3390/ph17010046)
Supplement: Supplementary file 1 [file pharmaceuticals-17-00046-s001.zip › Supplementary Figure S3.pdf]

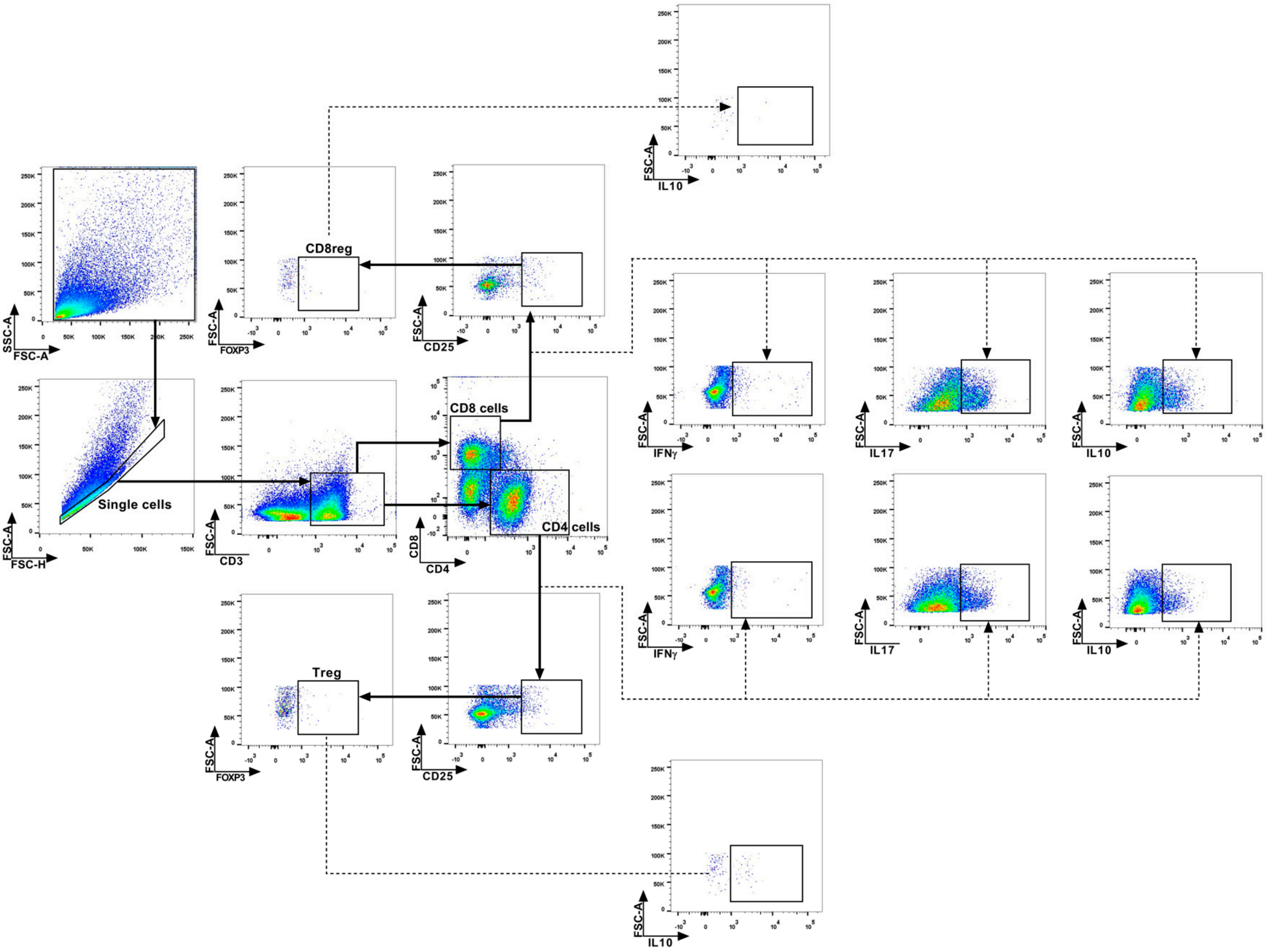

Supplementary Figure S3. A schematic representation of gating strategy for adaptative immune cells subpopulations isolated from spleen, lung and brain of mice and stained for IL-10, IFN-γ and IL-17 cytokine production, and corresponding dotplots of: IL-10^{+}CD4^{+} T cells, IFN-γ^{+}CD4^{+} T cells, IL-17^{+}CD4^{+} T cells; IL-10^{+}CD8^{+} T cells, IFN-γ^{+}CD8^{+} T cells, IL-17^{+}CD8^{+} T cells; and IL-10^{+}CD4^{+}Treg cells and IL-10^{+}CD8^{+}Treg cells
